# Speeding Towards the Goal That We Set Out

**DOI:** 10.1155/S0962935193000249

**Published:** 1993

**Authors:** Iván L. Bonta


					Editorial
Mediators of Inflammation, 2, 179 (1993)
Speeding Towards the Goal
That We Set Out
After 11/2 years of existence of Mediators ofInjqamma-
tion there is a 40% increase in manuscript submis-
sions. The performance of the Journal is such that
it is now included in Chemical Abstracts, Excerpta
Medica (EMBASE) and is listed in Current Contents.
All this is largely due to the occurrence of three
circumstances: (a) timely topics of the submitted
articles; (b) high standard of the published papers;
(c) rapidity of the review proces and the short time
from acceptance to publication.
However there is no room for complacency.
Hence the call-for-papers continues to be our slogan.
Further, if you find as a reader that our Journal
delivers important data to the scientific community,
please urge your librarian to subscribe, thus im-
proving the dissemination of the information and
thereby letting other colleagues profit from the
papers we are publishing.
When the Journal was launched, we set out to
be the most rapid prime forum of choice within the
field. We are approaching that goal, but have not
yet reached it. On behalf of my colleague Editors
and members of the Editorial Board, we encourage
you to help us in realizing that, Mediators of In-
jqammation obtains amongst scientific journals the
place which it deserves.
Ivtn L. Bonta
Editor-in-Chief
(C) 1993 Rapid Communications of Oxford Ltd Mediators of Inflammation. Vol 2.1993 179

				

